# Grading for grapevine downy mildew and feature extraction methods for predicting abaxial lesions from adaxial leaf images

**DOI:** 10.3389/fpls.2025.1688315

**Published:** 2025-10-22

**Authors:** Bohao Liu, Cuiling Li, Jianjun Hao, Jian Song, Haowei Liu, Hongwei Yan, Changyuan Zhai

**Affiliations:** ^1^ Intelligent Equipment Research Center, Beijing Academy of Agriculture and Forestry Sciences, Beijing, China; ^2^ College of Mechanical and Electrical Engineering, Hebei Agricultural University, Baoding, China; ^3^ Beijing PAIDE Science and Technology Development Co., Ltd., Beijing, China; ^4^ National Engineering Research Center of Intelligent Equipment for Agriculture (NERCIEA), Beijing, China

**Keywords:** grape downy mildew, classification, segmentation, adaxial/abaxial lesions, inversion

## Abstract

Grading grapevine downy mildew severity is essential for the precise application of pesticides. Since typical symptoms appear on the abaxial (underside) surface of grape leaves, and lesion area proportion determines severity, it is necessary to analyze lesion characteristics and develop adaxial-to-abaxial lesion inversion methods to build lightweight yet accurate grading models. This study proposes a comprehensive disease grading framework for grape downy mildew. First, a convolutional neural network (CNN)-based classification model is developed with specialized modules and coordinate attention to enhance feature extraction and semantic richness for improved lesion identification. Second, a novel K-Means++-CNN-Vote Consolidation lesion extraction method is introduced. In this framework, K-Means++ segments leaf sub-images, CNNs classify lesion types, and a voting mechanism consolidates results—addressing challenges posed by irregular lesion shapes and blurred boundaries. Finally, an abaxial lesion inversion framework is established by constructing a morphological feature mapping between the adaxial and abaxial surfaces, utilizing mapping functions and lesion generation techniques to infer the abaxial lesion distribution from the adaxial images. Experimental results showed disease grading accuracies of 82.16% (combined adaxial and abaxial), 79.74% (adaxial only), and 84.59% (abaxial only), with a model size of 5.08 MB. Lesion segmentation accuracies reached 89.29% (adaxial and abaxial), 76.92% (adaxial), and 64.47% (abaxial), while the adaxial-to-abaxial lesion inversion achieved an 80% similarity. This study provides methodological support for the online grading of grapevine downy mildew and offers a scientific basis for precise disease control.

## 1 Introduction

Grapes, known for their sweet-sour taste and rich nutritional content, are among the most widely consumed fruits globally (Tian et al., 2018; Liu et al., 2020). They represent a globally significant economic fruit crop, and the quality of grapes and their related processed products is vital to the stability of the agricultural economic chain (Sagar et al., 2025). Grapevines are frequently affected by diseases during growth, among which grape downy mildew—an airborne oomycete disease—poses a severe threat to leaf photosynthetic efficiency and fruit development (Ting et al., 2025; Wu et al., 2025). Infected fruits exhibit a deteriorated appearance and flavor, and fail to meet the standards for fresh consumption or winemaking. Grapevine downy mildew is highly epidemic, and once it erupts, it becomes difficult to control. Surveys indicate that outbreaks can reduce annual grape yields by 11.8%–25.9%, making them a critical biotic stress factor that limits grape quality improvement and economic development (Wang and Li, 2024). The epidemic period of grapevine downy mildew is closely linked to climatic conditions. In North China, symptoms typically emerge sporadically in July, presenting as irregular pale-yellow water-soaked spots on the adaxial leaf surface, with sparse, characteristic white downy mildew layers on the corresponding abaxial areas. The lesion edges on both sides are generally indistinct (Wang et al., 2022a). As autumn rains increase, the disease enters an explosive phase in August. Adaxial lesions rapidly enlarge, turning yellow-brown to dark brown, while abaxial mildew layers become dense and thick, transitioning from white to gray with a “white, frost-like” appearance, yet still retaining blurred edges (Liu et al., 2022). By September, late-stage symptoms manifest as irregular desiccation cracks and perforations due to tissue necrosis on the adaxial surface, whereas abaxial surfaces show sparse grey mildew spots that significantly reduce photosynthesis (Yu et al., 2024). The current integrated management of grapevine downy mildew relies primarily on chemical control (Wang et al., 2022). However, extensive chemical spraying methods result in excessive pesticide residue and environmental pollution, highlighting the need for severity grading to enable differentiated precision control.

In recent years, vision-based methods for detecting grape leaf diseases have been extensively studied, gradually replacing manual identification of downy mildew. Traditional machine vision techniques primarily rely on image preprocessing and machine learning methods, such as support vector machines, random forest (RF), and K-means clustering algorithms, for automated classification. For example, Li et al. (2011) selected 30 diseased leaves with clean surfaces, applied the K-Means clustering algorithm to segment leaf and lesion areas, accurately calculated the lesion-to-leaf area ratio using pixel statistics, and assigned severity grades based on classification standards, achieving an accuracy of 93.33%. These methods are based on manually defined rules, require smaller sample sizes, lower annotation demands, and offer interpretability. They are suitable for environments with uniform lighting and simple backgrounds. However, they depend heavily on manual feature extraction, requiring domain expertise to define relevant characteristics and understand specific research objectives (Kumar and Sachar, 2023). Moreover, their effectiveness deteriorates significantly when the detection environment changes (Xu et al., 2025). Deep learning approaches, which leverage end-to-end feature learning, have become a research hotspot. These approaches employ numerous advanced models (He et al., 2016; Joseph et al., 2016; Sandler et al., 2018; Ma et al., 2018; Tan and Le, 2021) and are remarkably accurate in disease prediction. These network architectures are capable of recognizing intricate patterns and characteristics within datasets, thereby significantly improving prediction accuracy (Zhang et al., 2024). For instance, He et al. (2022) proposed an improved ResNet50 grading network that classified grapevine downy mildew into four stages—healthy, pre-infection, mid-infection, and late infection—with an accuracy of 99.92%. Although this method utilizes a single convolutional neural network (CNN) to determine infection stages, its large parameter size limits deployment on edge devices. It does not align with national severity grading standards. Zohaib et al. (2025) employed an improved You Only Look Once version 7 algorithm to distinguish between unhealthy leaves, healthy leaves, and grape cluster bags, achieving an accuracy of 73.7%. Yang and Qiu (2024). proposed the YOLOv8s-grape model, which integrates multiple enhancements to achieve efficient, high-precision grape detection with improved mAP and reduced computational costs. Fu et al. (2024) proposed the MHDI-DETR model, a lightweight RT-DETR-based architecture achieving high accuracy in grape leaf disease detection with significantly reduced computational complexity. While this object detection approach locates lesions and detects disease presence, it requires manual lesion annotation in advance and does not assess severity levels according to national standards. These studies indicate that current vision-based methods for grapevine downy mildew detection rely on manual feature engineering in traditional approaches for simple scenarios, whereas deep learning CNN-based classification and object detection methods achieve high accuracy but lack fine-grained severity grading. Therefore, there is a pressing need for a lightweight and precise model capable of grading adaxial/abaxial lesion severity to achieve accurate disease classification and enable precision pesticide application.

The domestic standard for pesticide application against grapevine downy mildew follows China’s Guidelines for Field Efficacy Trials of Pesticides, Part 122: Fungicides against Grapevine Downy Mildew (Standard No.: GB/T 17980.122-2004), which classifies disease severity based on ​leaf lesion coverage ​ and provides corresponding spray protocols. ​Lesion segmentation is a critical step for accurately implementing this standard. Only through accurate segmentation can detailed parameters, such as lesion size, shape, and distribution characteristics, be obtained, offering robust support for scientific grading and control strategies. Current segmentation methods can be categorized into two main types: machine-learning-based and deep learning-based approaches (Ji and Wu, 2022). For instance, Li et al. (2022) utilized the K-Means clustering algorithm to segment grape leaves into subregions and trained a random forest classifier with minimal dataset labeling, achieving a segmentation accuracy of 63.32%. This strategy prevents complex, time-consuming manual annotation, but suffers from limited precision. Conversely, Zhang et al. (2025) proposed the ABLSS model, which integrates deep and broad learning to achieve high-accuracy, efficient grape leaf disease identification with improved recognition speed and segmentation performance. Wu (2024) adopted a ​two-step segmentation strategy​ using ​U-Net networks to separately segment leaves and lesions, achieving ​a disease classification accuracy of 93.3%. Unlike Wu (2023), Xue (2023) designed the IN-UNet network for the complete semantic segmentation of grape leaves and lesions, achieving a pixel-wise segmentation accuracy of 84.79% and enabling grading that aligns with national standards. Although deep learning methods yield higher accuracy and broader applicability, they often require ​labor-intensive expert annotations. For lesions with ​blurred boundaries or dense clustering, manual labeling becomes particularly challenging (Li et al., 2022). Tardif et al. (2023) reported that accurately delineating diseased boundaries in grapevine images can require ​up to 20 min per annotation, and Ahmad et al. (2023) emphasized that precise symptom shape labeling in segmentation tasks constitutes significant labor. To address this, we propose a k-Means++ + CNN + visual classification (K-CNN-VC) lesion segmentation method based on our grading network model, which generates grapevine downy mildew lesion labels using only simple classification annotations, thereby bypassing the challenges associated with complex, time-consuming, and ambiguous labeling of difficult samples.

The characteristic symptoms of grapevine downy mildew manifest predominantly on the abaxial leaf surface, and disease severity grading typically relies on the proportion of the abaxial lesion area. However, under natural conditions, grape leaves predominantly exhibit an adaxial-facing orientation. Manual detection requires leaf flipping, which entails high labor intensity and subjectivity (Hernández et al., 2024). Similarly, images captured by inspection-spraying robots primarily feature adaxial surfaces, leading to detection inaccuracies. Consequently, spraying decisions derived from such data may result in dosage deviations, increasing the risk of fungicide resistance and potential environmental contamination due to over-application (Jin et al., 2022). Therefore, developing adaxial-to-abaxial lesion inversion methods is crucial. Zhang et al. (2019) acquired both adaxial and abaxial images of wheat ears, processed them through grayscale conversion and binarization, calculated the lesion-to-ear area ratio, and graded the severity of Fusarium head blight based on national standards. Although this method integrates adaxial-abaxial disease information, it does not separately compare severity levels against national criteria. Yang (2020) utilized ResNet50 to identify pear leaf diseases (brown spots, black spots, and rust) on adaxial and abaxial surfaces, achieving recognition accuracies of 96.67% and 96.19%, respectively. Wayama et al. (2024) collected adaxial/abaxial images of diseased leaves from tomatoes, strawberries, cucumbers, and eggplants, employing EfficientNet for classification. The model achieved 95.2% accuracy in consistent environments but only 36.5% in varying environments. This study addressed cross-environment generalizability but ignored adaxial-abaxial symptom differences. These approaches overlook the symptomatic distinctions and correlations between the adaxial and abaxial surfaces, limiting their research scope and applicability. Therefore, it is essential to investigate whether adaxial and abaxial lesion severity align quantitatively, analyze correlations when inconsistencies exist, and leverage these relationships to infer abaxial lesions from adaxial images, thereby obtaining accurate disease severity grades and achieving adaxial-to-abaxial lesion inversion.

Although progress has been made in grading the severity of grape downy mildew, current research primarily focuses on disease type identification or lesion morphology extraction and description, with limited attention given to fine-grained severity grading. Significant challenges remain, including the fine-grained recognition of disease severity levels via CNN methods, annotation difficulties with lesions that have blurred boundaries in deep learning, a quantitative comparative analysis of adaxial and abaxial lesions, and the inversion of abaxial lesions from adaxial grapevine downy mildew images. The specific objectives of this study are as follows:

1. To construct a lightweight and precise adaxial/abaxial lesion grading network for grapevine downy mildew by designing a cross-receptive-field fusion module that integrates regular convolution and depthwise separable convolution to enhance semantic richness, while incorporating a coordinate attention (CA) mechanism to strengthen the feature extraction capabilities for adaxial/abaxial lesions.
2. To propose the K-CNN-VC lesion segmentation method for precise quantitative analysis of lesion areas. This method segments leaf sub-images via K-Means++, classifies them using the grading network, and consolidates them through voting, thereby addressing the time-consuming and imprecise manual annotation of complex lesions.
3. To conduct a quantitative comparative analysis of adaxial and abaxial lesions in grapevine downy mildew to establish morphological mapping relationships between the two surfaces.
4. To develop a vision perception-based theory for adaxial-abaxial lesion inversion, achieving end-to-end mapping from adaxial lesion features to abaxial lesion morphologies by constructing a morphological mapping model, thereby providing a novel scientific basis for precision spraying.

## 2 Materials

### 2.1 Data acquisition

**Figure 1:**
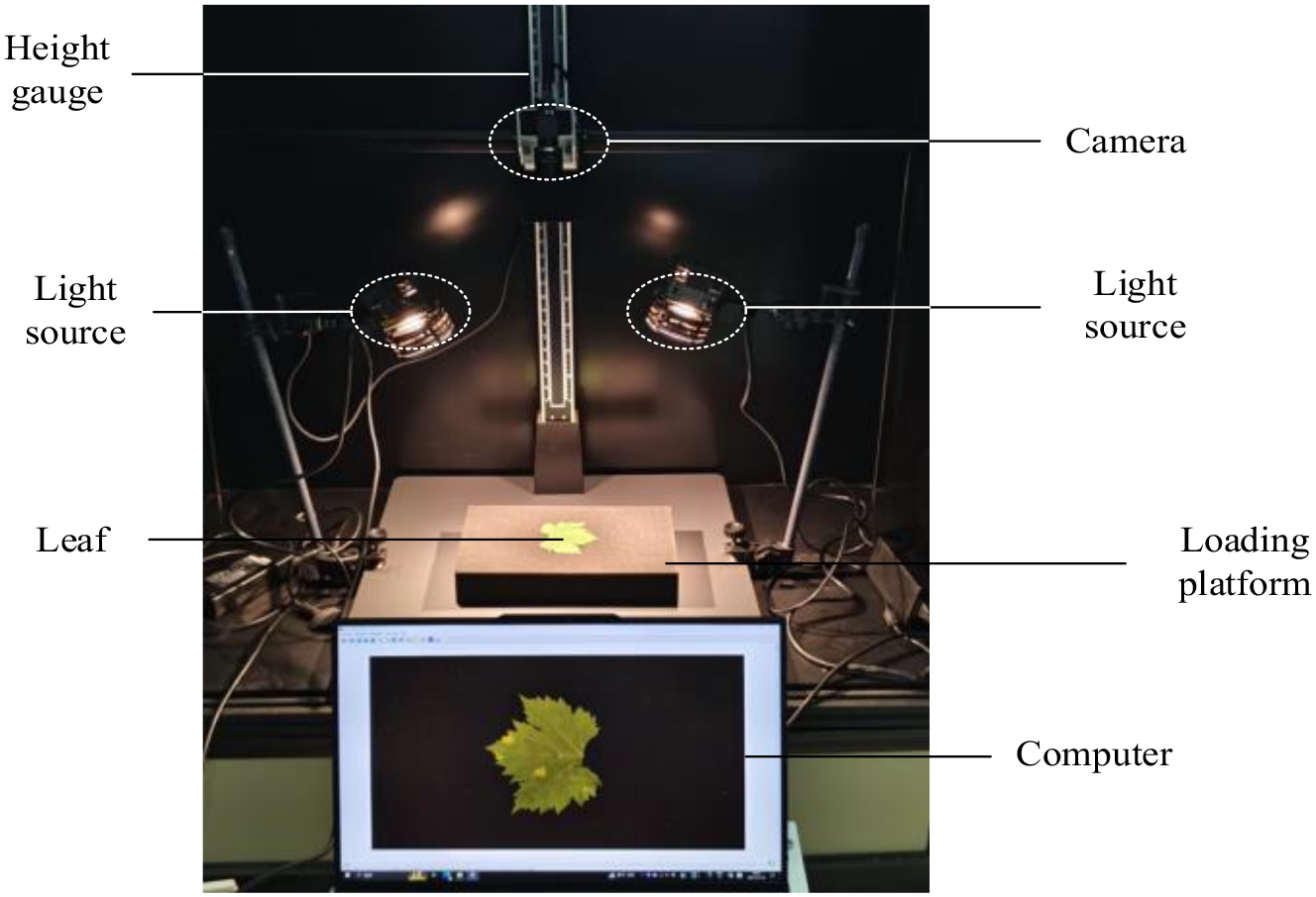
Image acquisition system.

To investigate lesion characteristics and develop grading methods for grape downy mildew, we collected RGB images of detached grape leaves infected with the disease. Early-stage disease images were collected at the Xiaotangshan National Precision Agriculture Research Demonstration Base in Beijing, and late-stage images were collected at the Chateau Bolongbao Winery in Fangshan, Beijing. The image-acquisition system consisted of an MV-SUA505GM camera equipped with an MS3M008 lens at a working distance of 50 cm. The resulting dataset comprised downy mildew leaf images from five grape varieties: Wild Grape, Kyoho, White Grape, Ningxia Wild Grape, and Cabernet Sauvignon. A total of 674 RGB images were collected, including 430 early-stage and 244 late-stage images. Each image had an original resolution of 2592 × 2048 pixels. **Figure 1** shows an overview of the image acquisition system.

### 2.2 Data processing and augmentation

The original images were initially center-cropped to a resolution of 1024 × 1024 pixels and subsequently downsampled to 512 × 512 pixels. To address the challenges posed by an insufficient number of grapevine downy mildew leaf samples and imbalanced distribution of severity levels, we applied data augmentation techniques (González et al., 2025) to enhance model generalization and reduce overfitting during training. Using geometric transformations such as rotation and flipping, we expanded the dataset to a total of 5,392 images, forming the Grape Downy Mildew Image Dataset (GDCData). This dataset was divided into training, validation, and test sets in a 7:2:1 ratio. **Table 1** shows the representative augmentation results, where z0 denotes adaxial healthy leaves, z1–z7 represents adaxial lesion severity grades, f0 indicates abaxial healthy leaves, and f1–f7 signifies abaxial lesion severity grades.

**Table 1:** Sample distribution of the GDCData dataset for grape downy mildew.

Level	Z0	Z1	Z3	Z5	Z7	F0	F1	F3	F5	F7	Total
Expand before	40	94	126	50	27	40	96	122	53	26	674
Expanded	320	752	1008	400	216	320	768	976	424	208	5392

## 3 Methods

### 3.1 Classification method

To balance classification accuracy and computational efficiency, we designed a custom lightweight grading model, GDCNet, as detailed in **Table 2**, for grapevine downy mildew severity classification. This network architecture primarily consists of two components: cross-receptive field fusion (CRFF) and attention modules. Downsampling operations employ 3 × 3 convolution kernels with a stride of two. The CRFF module integrates depthwise separable convolution and pointwise convolution, enabling shortcut connections that allow the network to deepen effectively while preserving and enhancing discriminative lesion features. The attention module implements parallel attention mechanisms, including CA, to focus on spatially local features relevant to grape leaf lesions. During training and interference, input images were resized to 512 × 512. At the end of the output, an adaptive average pooling layer consolidated feature maps to 1 × 1 × C dimensions. Finally, a two-layer fully connected classifier with a softmax activation function was used to categorize severity levels based on learned features.

**Table 2:** GDCNet network structure table.

Input	Operator	Exp size	Output	CA	Stride
512^2^×3	SCDCA, 3×3	32	32	0	1
512^2^×32	SCDCA, 3×3	64	32	0	2
256^2^×32	SCDCA, 3×3	64	32	0	1
256^2^×32	SCDCA, 3×3	64	32	1	1
256^2^×32	SCDCA, 5×5	128	64	0	2
128^2^×64	SCDCA, 5×5	128	64	0	1
128^2^×64	SCDCA, 5×5	128	64	1	1
128^2^×64	SCDCA, 3×3	256	128	0	2
64^2^×128	SCDCA, 3×3	256	128	1	1
64^2^×128	SCDCA, 3×3	256	128	0	1
64^2^×128	SCDCA, 3×3	256	128	1	1
64^2^×128	SCDCA, 3×3	256	128	0	1
64^2^×128	SCDCA, 3×3	256	128	1	1
64^2^×128	SCDCA, 3×3	256	128	0	1
64^2^×128	SCDCA, 3×3	256	128	1	1
64^2^×128	SCDCA, 5×5	512	256	0	2
32^2^×256	SCDCA, 5×5	512	256	0	1
32^2^×256	SCDCA, 5×5	512	256	1	1
32^2^×256	AdaptiveAvgPool	–	256	–	–
1^2^×256	Linear	–	1280	–	–
1^2^×1280	Hardswish	–	1280	–	–
1^2^×1280	Dropout 0.2	–	1024	–	–
1^2^×1024	Linear	–	num_classes	–	–

#### 3.1.1 Skip-connected depthwise coordinate attention model

**Figure 2:**
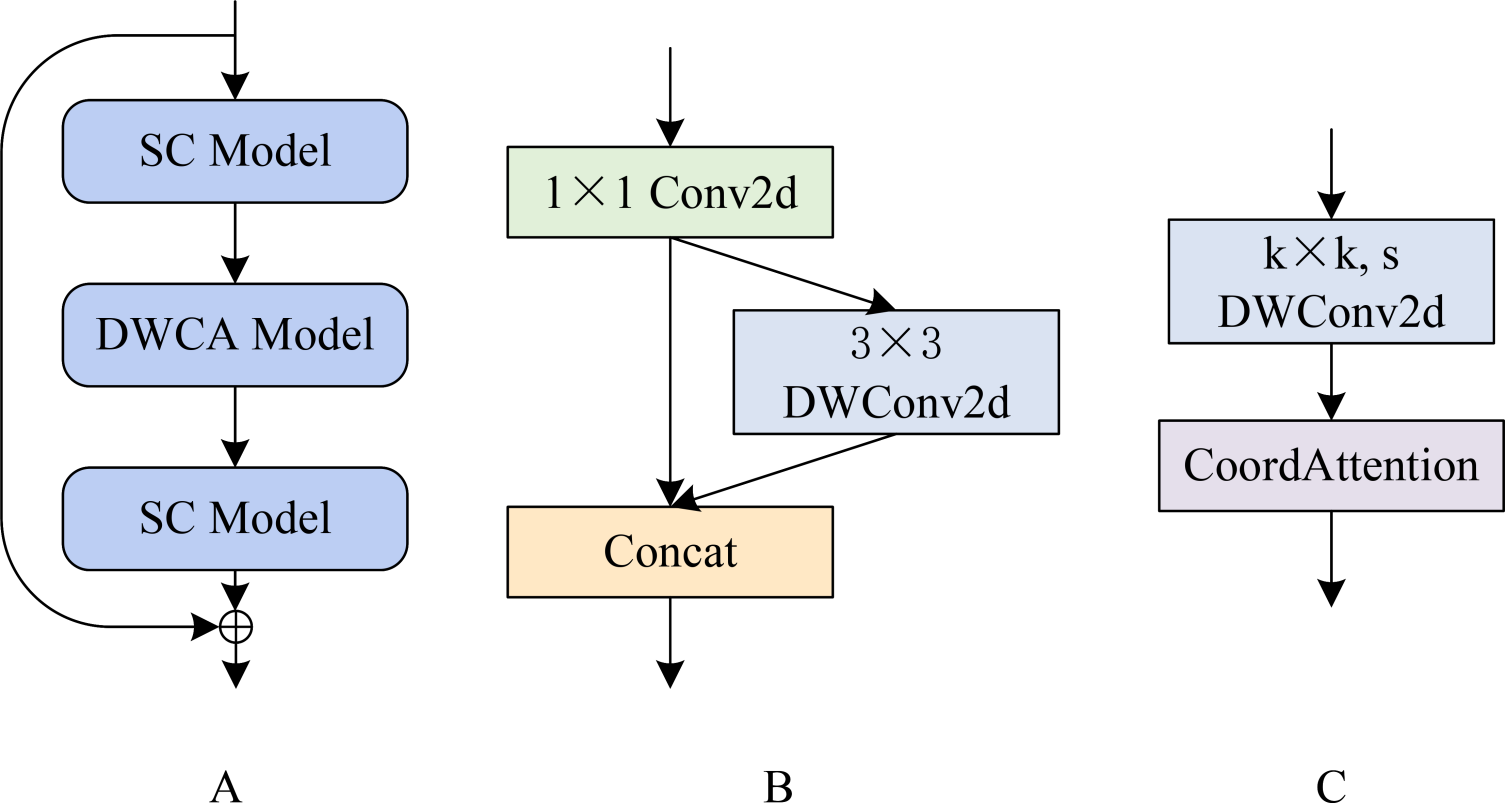
SCDCA module structure diagram. **(A)** shows the SCDCA Model; **(B)** shows the SC Model; **(C)** shows the DWCA Model.

**Figure 2A** shows that the SCDCA Model comprises a series of stacked modules. The processing pipeline involves three main components executed in sequence: a separable convolution (SC) module (**Figure 2B**), followed by a depth-wise convolution with depth-wise coordinate attention (DWCA) module (**Figure 2C**), and a final SC Model. A skip connection is established by directly connecting the input of the first SC module to the output of the final SC module. This architectural design integrates modular processing with skip connections, effectively leveraging complementary advantages to enhance the capacity of the model for learning and processing complex features.

The SC Model begins by applying a 1 × 1 two-dimensional convolution to the input feature map. The resulting feature map is then split into two parallel processing paths: one path bypasses further processing directly, and the other path is processed through a 3 × 3 depthwise separable convolution, as described by Tang et al. (2020). These outputs from both paths are concatenated to form the final output of the SC module. This dual-path architecture facilitates multiscale feature extraction and fusion, thereby enhancing the ability of the network to represent and differentiate fine-grained lesion features.

The DWCA module initiates feature extraction using a k × k depth-wise separable convolution with a stride of s. The resulting features are passed through a CA mechanism, which captures inter-channel relationships while embedding spatial directional information. This enables the model to focus on key lesion regions, enhancing the sensitivity and accuracy of spatial feature extraction.

#### 3.1.2 Coordinate attention

Coordinate Attention is an efficient attention mechanism proposed by Hou et al. (2021) that enhances feature representation by embedding spatial information into channel attention. Unlike traditional channel attention, which ignores positional context, Coordinate Attention divides two-dimensional global pooling into dual one-dimensional feature encodings along the horizontal and vertical axes, thus preserving precise spatial location cues. A detailed analysis, including relevant formulas, is presented below.

##### 3.1.2.1 Coordinate information embedding

Given an input feature map $X\in R^{H\times W\times C}$ , the attention mechanism computes two direction-specific aggregated descriptors:

​Horizontal Pooling: For channel c and height h, the average across the width is calculated as:


$$z_{h}\left(c,h\right)=\frac{1}{W}\sum_{w=1}^{W}X\left(c,h,w\right)$$

Output:


$$z_{h}\in R^{C\times H}$$

​Vertical Pooling: For channel c and width w, the average across the height is computed as:


$$z_{w}\left(c,w\right)=\frac{1}{H}\sum_{h=1}^{H}X\left(c,h,w\right)$$

Output:


$$z_{h}\in R^{C\times w}$$

##### 3.1.2.2 CA generation

The pooled features are concatenated and transformed to generate attention weights:

​Concatenation and Compression: Concatenate $z_{h}$ and $z_{w}$ along the spatial dimension to form $z\in R^{C\times \left(H+W\right)}$ . Apply a 1×1 convolution for dimensionality reduction, followed by activation:


$$f=\delta \left(Conv1x1\left(z\right)\right),\;f\in R^{C/r\times \left(H+W\right)}$$

where r denotes the reduction ratio and δ represents the ReLU function.

​Splitting and Reconstruction: Split f into $f_{h}\in R^{C/r\times H}$ and $f_{w}\in R^{C/r\times W}$ , then restore the channel dimension via 1 × 1 convolutions:


$$g_{h}=Conv1x1\left(f_{h}\right),g_{w}=Conv1x1\left(f_{w}\right)$$

​Attention Weights Generation: The output tensors ( $g_{h}$ and $g_{w})\;$ are passed through sigmoid activation​:


$$a_{h}=\sigma \left(g_{h}\right)\in R^{C\times H},a_{w}=\sigma \left(g_{w}\right)\in R^{C\times W}$$

##### 3.1.2.3 Applying attention weights

The attention weights are broadcast to match the dimensions of the input feature map and applied through element-wise multiplication:


$$Y\left(c,h,w\right)=X\left(c,h,w\right)\cdot a_{h}\left(c,h\right)\cdot a_{w}\left(c,w\right)$$

Broadcast the attention weight $a_{h}$ to dimensions $R^{C\times H\times 1}$ and $a_{w}$ ​ to $R^{C\times 1\times W}$ . Then, compute their element-wise product to derive the combined weight $R^{C\times H\times W}$ .

By decomposing the spatial dimensions, this method preserves precise, coordinate-aware features while significantly enhancing the localization capability of the model for critical regions. The use of one-dimensional convolutions minimizes computational load, making the mechanism highly suitable for lightweight network designs.

### 3.2 K-CNN-VC segmentation method

**Figure 3:**
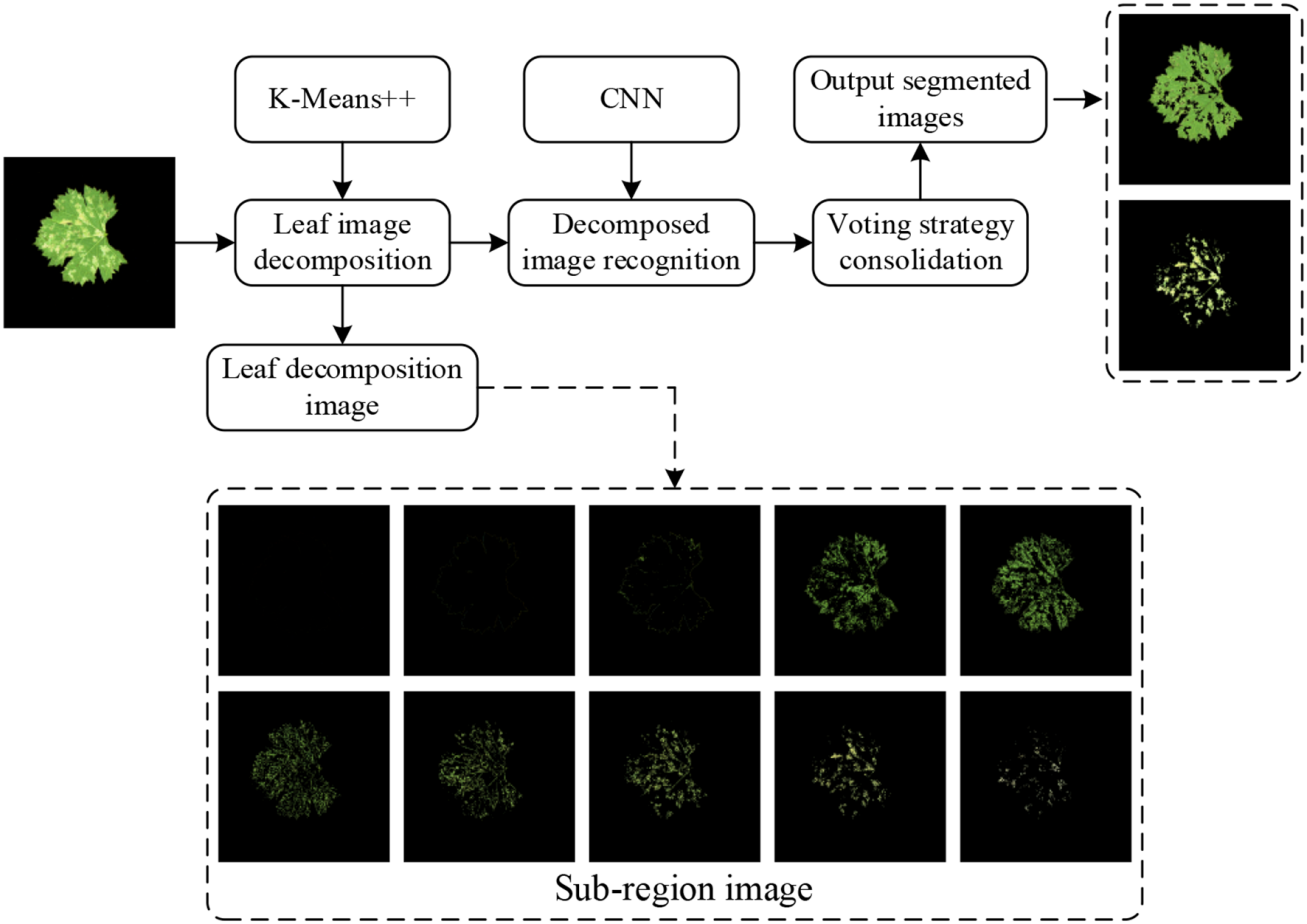
K-CNN-VC segmentation flowchart.

Owing to the morphological complexity of grapevine downy mildew symptoms, manual pixel-level annotation is both labor-intensive and resource-demanding, making direct label-based training infeasible. Traditional image processing or unsupervised learning methods require manual identification of extractable features across massive datasets to design segmentation rules. Furthermore, variations in leaf brightness, uneven illumination, and divergent lesion characteristics across severity levels render manual feature extraction extremely challenging. To overcome these challenges, we propose a hybrid lesion segmentation method that combines K-means++ clustering with CNN-based classification (K-CNN-VC method), enabling efficient and automated lesion area identification with minimal supervision. Unsupervised learning first decomposes images into sub-regions with homogenous color features, while supervised training classifies these sub-regions. Issues such as potential misclassifications are corrected using voting consolidation. Finally, sub-regions are recombined to identify lesions and leaf areas. This approach indirectly applies supervised learning to automated lesion feature extraction, as illustrated in **Figure 3**.

The K-Means++ algorithm segments leaf images into multiple sub-regions. These sub-regions are then classified using a CNN. They are recombined to identify lesion areas and leaf regions. The grape downy mildew lesion extraction process comprises three stages: leaf image decomposition, recognition of the decomposed image, and image recombination.

### 3.3 Inversion model

**Figure 4:**
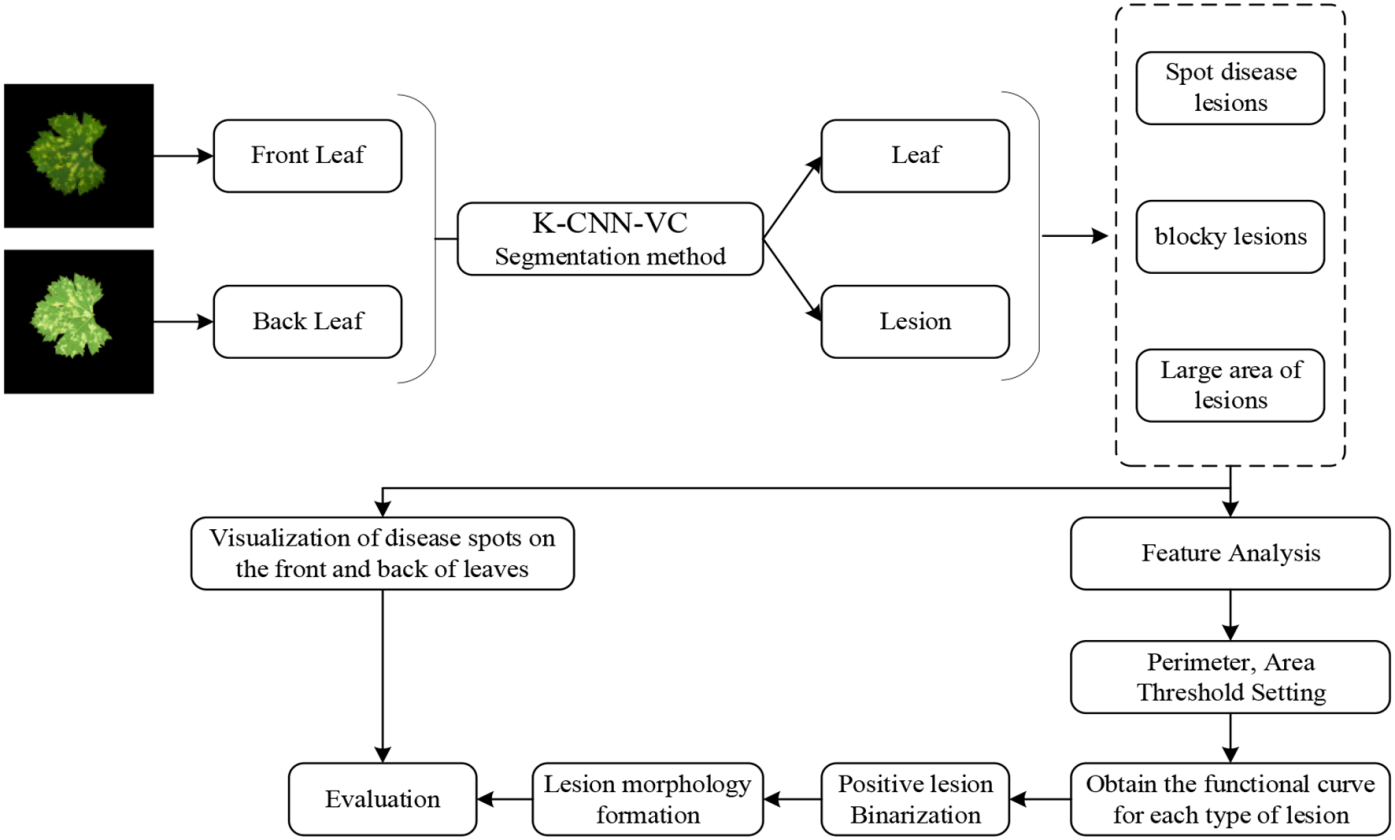
Flowchart of the inversion method. Based on the established mappings, the abaxial lesion shapes were generated using morphological operations. If both the perimeter and area of the abaxial lesion exceeded the adaxial lesion, a dilation operation was performed on the adaxial contour. If both were smaller, an erosion operation was applied, ultimately generating an abaxial lesion image. This regression-guided morphological generation algorithm enables biologically consistent modeling of lesion propagation patterns from adaxial to abaxial leaf surfaces.

To simulate the progression and visibility of grape downy mildew lesions from the adaxial to the abaxial leaf surface, we propose an adaxial-to-abaxial lesion inversion method, as illustrated in **Figure 4**. The K-CNN-VC segmentation method decomposes input images into five distinct classes: background, adaxial leaf, abaxial leaf, adaxial lesions, and abaxial lesions. Through empirical observation, adaxial lesions were categorized into three morphological types: dot-shaped, patch-shaped, and large-scale. For each segmented section, we extracted leaf and lesion contours from the segmented results. Then, the contour perimeter and enclosed area of the lesions were calculated. Using the perimeter-area pairs, threshold-based classification was applied to map adaxial lesions to the corresponding abaxial lesion types. We established lesion-type-specific regression curves relating adaxial/abaxial areas, as well as contour perimeters versus enclosed areas. Finally, based on the established mappings, abaxial lesion morphologies were generated using morphological operations.

### 3.4 Experimental equipment and performance indicators

The experiments were conducted on a workstation configured as follows: Intel^®^ Core™ i9-12900K CPU@3.70 GHz, 32GB RAM, NVIDIA GeForce RTX 4070Ti Super 16GB GPU, and 64-bit Windows OS. The models were built using PyTorch 2.0.1 (a deep-learning framework). After extensive tuning, the final training hyperparameters were set as follows: Optimizer: Adam.Learning rate: Cosine decay with initial_lr = 1e-4 and min_lr = 1e-7. Momentum: 0.9. Weight decay: 1e-2. Batch size: 8. Epochs: 100. Drop path rate: 0.1 (to prevent overfitting). The grape downy mildew dataset was partitioned into training, validation, and test sets in a 7:2:1 ratio.

The evaluation metrics for the grading tasks include accuracy (Acc), mean precision (mP), mean recall (mR), parameter count, inference speed (FPS) (Zhang and Mu, 2024) and confusion matrix. Additionally, the total number of parameters and floating-point operations was used to measure the model size and computational complexity (Wang et al., 2023; Tan et al., 2025). Acc, mP, and mR were calculated as follows:


$$Acc=\frac{TP+TN}{TP+TN+FN+FP}$$

$$mP=\frac{1}{k}\frac{TP}{TP+FP}$$

$$mR=\frac{1}{k}\frac{TP}{TP+FN}$$

## 4 Results

### 4.1 Hierarchical model validation

**Figure 5:**
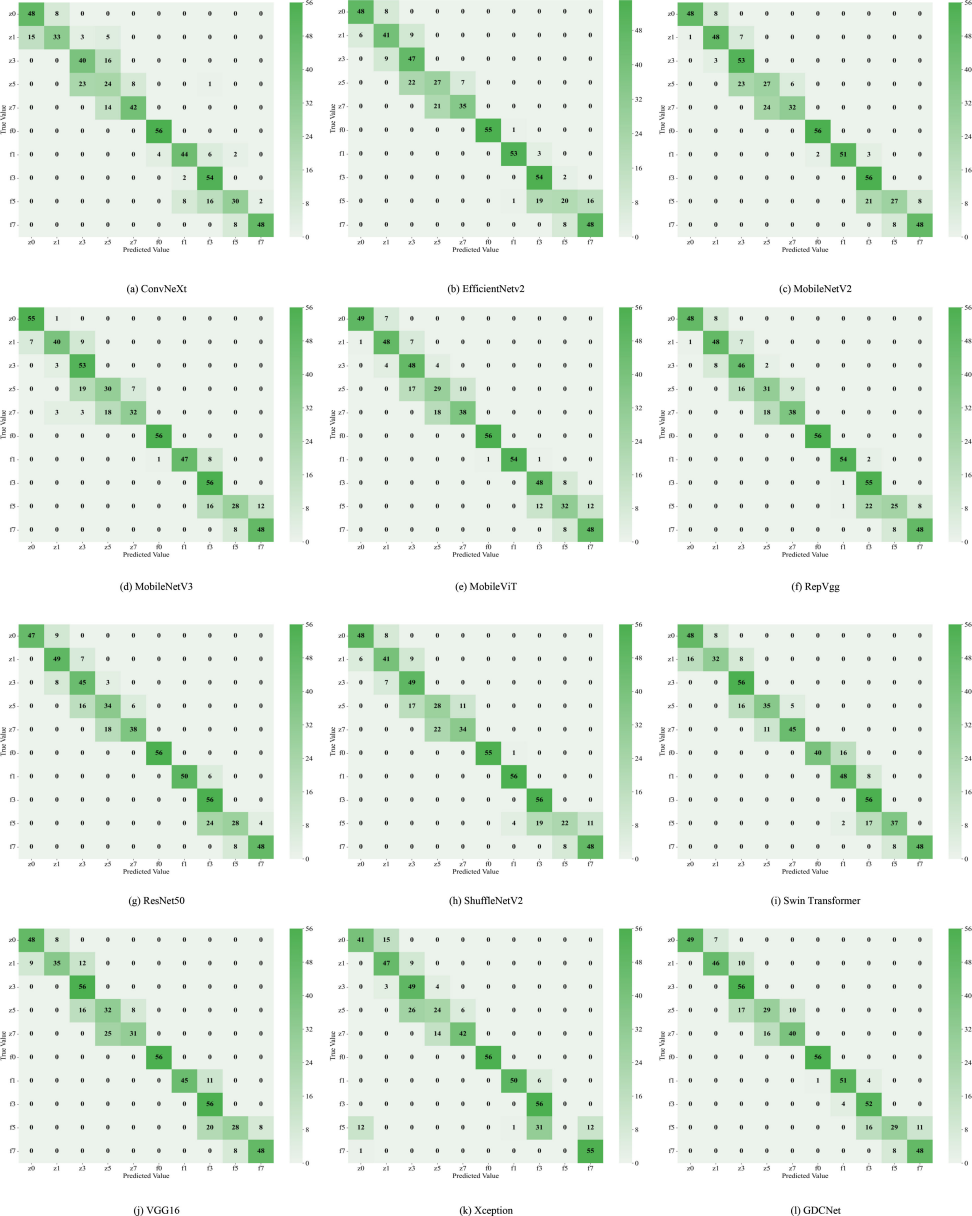
Twelve types of network classification confusion matrices. **(a)** displays the classification confusion matrix of the ConvNeXt network; **(b)** displays the classification confusion matrix of the EfficientNetV2 network; **(c)** displays the classification confusion matrix of the MobileNetV2 network; **(d)** displays the classification confusion matrix of the MobileNetV3 network; **(e)** displays the classification confusion matrix of the MobileViT network; **(f)** displays the classification confusion matrix of the RepVgg network; **(g)** displays the classification confusion matrix of the ResNet50 network; **(h)** displays the classification confusion matrix of the ShuffleNetV2 network; **(i)** displays the classification confusion matrix of the Swin Transformer network; **(j)** displays the classification confusion matrix of the VGG16 network; **(k)** displays the classification confusion matrix of the Xception network; **(l)** displays the classification confusion matrix of the GDCNet network.

To evaluate the effectiveness of our GDCNet grading model, we conducted a comparative analysis involving twelve vision models on tasks involving the detection of adaxial/abaxial leaf health states (z0/f0), classification of multiple disease severity levels, and overall model performance (Acc, mP, mR, giga floating point operations (GFLOPS), parameter scale P/M, and inference speed). The results are presented in **Tables 3**, **4** and **Figure 5**. Based on category-specific accuracy, ResNet50, RepVGG, and VGG16 achieved 100% precision in detecting adaxial healthy leaves. Additionally, ResNet50, RepVGG, ShuffleNetV2, and others also achieved perfect classification, indicating a strong discriminative capability for disease-free samples. Conversely, Xception performed poorly on high-severity grades (such as z3 and f5), with complete failure in f5. This reflects that its feature extraction mechanism is inadequate for complex disease morphologies. The specialized model GDCNet achieved exceptional performance in z0 and z1 categories (early-stage adaxial lesions), surpassing most general models in fine-grained early disease classification. This indicates that its optimized design effectively enhances fine-grained classification for early-stage disease features. Lightweight models, such as MobileNetV3_S and MobileViT, achieved 100% accuracy in F1, revealing sensitivity to superficial features of low-grade infections. Conversely, Swin_Transformer and GDCNet performed relatively better in severe disease grades.

**Table 3:** Classification accuracy of twelve models on lesion grades.

Model	Z0/%	Z1/%	Z3/%	Z5/%	Z7/%	F0/%	F1/%	F3/%	F5/%	F7/%
ConvNeXt_T	76.19	80.49	60.61	40.68	84.00	93.33	81.48	70.13	75.00	96.00
EfficientNetv2_S	85.71	73.21	83.93	48.21	62.50	98.21	94.67	96.43	35.71	85.71
MobileNetV2	97.96	81.36	63.86	52.94	84.21	96.55	100	70.00	77.14	85.71
MobileNetV3_S	88.71	85.11	63.10	62.50	82.05	98.25	100	70.00	77.78	80.00
MobileViT	98.00	81.36	66.67	56.86	79.17	98.25	100	78.69	66.67	80.00
RepVgg	97.96	85.00	66.67	60.78	80.85	100	96.43	69.62	75.76	85.71
ResNet50	100	74.24	66.18	61.82	86.36	100	100	65.12	77.78	92.31
ShuffleNetV2	88.89	73.21	65.33	56.00	78.86	100	91.80	74.67	73.33	81.36
Swin_Transformer	75.00	80.00	70.00	76.09	90.00	100	72.73	69.14	82.22	100
VGG16	84.21	81.40	66.67	56.14	79.49	100	100	64.37	77.78	85.71
Xception	75.93	72.31	58.33	5714	87.50	100	98.04	60.22	0	82.09
GDCNet	100	86.79	67.47	64.44	80.00	98.25	92.73	72.22	78.38	81.36

**Table 4:** Performance comparison of twelve models.

Model	Acc/%	Mp/%	Mr/%	GFLOPS/G	P/m	Speed/ms
ConvNeXt_T	74.82	75.79	74.82	46.528	27.806	0.93
EfficientNetV2_S	76.43	76.85	76.43	30.243	20.190	1.28
MobileNetV2	79.64	80.97	79.64	3.409	2.237	0.46
MobileNetV3_S	79.46	80.75	79.46	0.630	1.528	0.40
MobileViT	80.36	80.57	80.36	3.005	0.954	0.52
RepVgg	80.18	80.88	80.18	15.981	9.109	0.74
ResNet50	80.54	82.38	80.54	43.172	23.529	0.93
ShuffleNetV2	78.04	78.02	78.04	0.455	0.352	0.38
Swin_Transformer	79.46	81.52	79.46	8.742	27.504	1.28
VGG16	77.68	79.58	77.68	160.596	134.302	5.99
Xception	75.00	69.15	75.00	48.490	8.454	0.52
GDCNet	81.43	82.16	81.43	2.441	1.245	0.56

For the overall metrics, GDCNet ranked first with 81.43% Acc, 82.16% mP, and 81.43% mR. Its computational cost and parameter scale were significantly lower than those of heavyweight models, such as ResNet50 and VGG16, whereas its inference speed was comparable to that of MobileNetV3_S, demonstrating a balanced optimization of accuracy and efficiency. Among the lightweight models, ShuffleNetV2 achieved an accuracy of 78.04% with minimal GFLOPS and parameters, making it suitable for ultra-lightweight deployment. MobileViT enhances feature representation while retaining speed advantages through a hybrid vision transformer architecture, validating the effectiveness of cross-modal fusion. Traditional convolutional models, such as VGG16, excel in f0/f1 categories, but suffer from low practicality due to high computational overhead (5.99 ms inference latency).

Collectively, GDCNet exhibited optimal comprehensive performance in multigrade grape downy mildew detection via customized feature learning and a lightweight design. Among the general models, ResNet50 showed robustness for healthy samples and partial disease grades, fitting scenarios with relaxed computational constraints. Conversely, MobileNetV3_S and ShuffleNetV2 offer advantages in speed and low-grade disease detection, but require improvement in feature abstraction for advanced infections. The inefficiency of Xception requires structural adaptations for multiscale, heterogeneous features of grape leaf diseases. In contrast, the accuracy bottleneck in high-severity categories (for example, z5) across models highlights future research priorities: which is to enhance complex lesion representation through attention mechanisms or cross-layer feature fusion techniques.

### 4.2 Results and Analysis of K-CNN-VC Segmentation Method

To construct a robust segmentation dataset for grape downy mildew, we employed the proposed K-CNN-VC segmentation method on a total of 6,740 leaf images. The pipeline applied K-means++ clustering to decompose each image into sub-regions based on consistent color features, constructing the GDSData segmentation dataset. **Table 5** details the categorization results. The training, validation, and test sets were distributed in a 7:2:1 ratio, where ​0 denotes background, ​1 denotes adaxial leaf surface, ​2 denotes abaxial leaf surface, ​3 denotes adaxial lesions, ​4 denotes abaxial lesions, ​5 denotes adaxial veins, ​6 denotes abaxial veins, ​7 denotes lesions and veins, and ​8 denotes leaves and lesions.

**Table 5:** Sample distribution of the GDSData partitioned dataset.

Category	0	1	2	3	4	5	6	7	8	Total
Quantity	1740	1786	1643	587	520	94	91	194	85	6740

**Figure 6:**
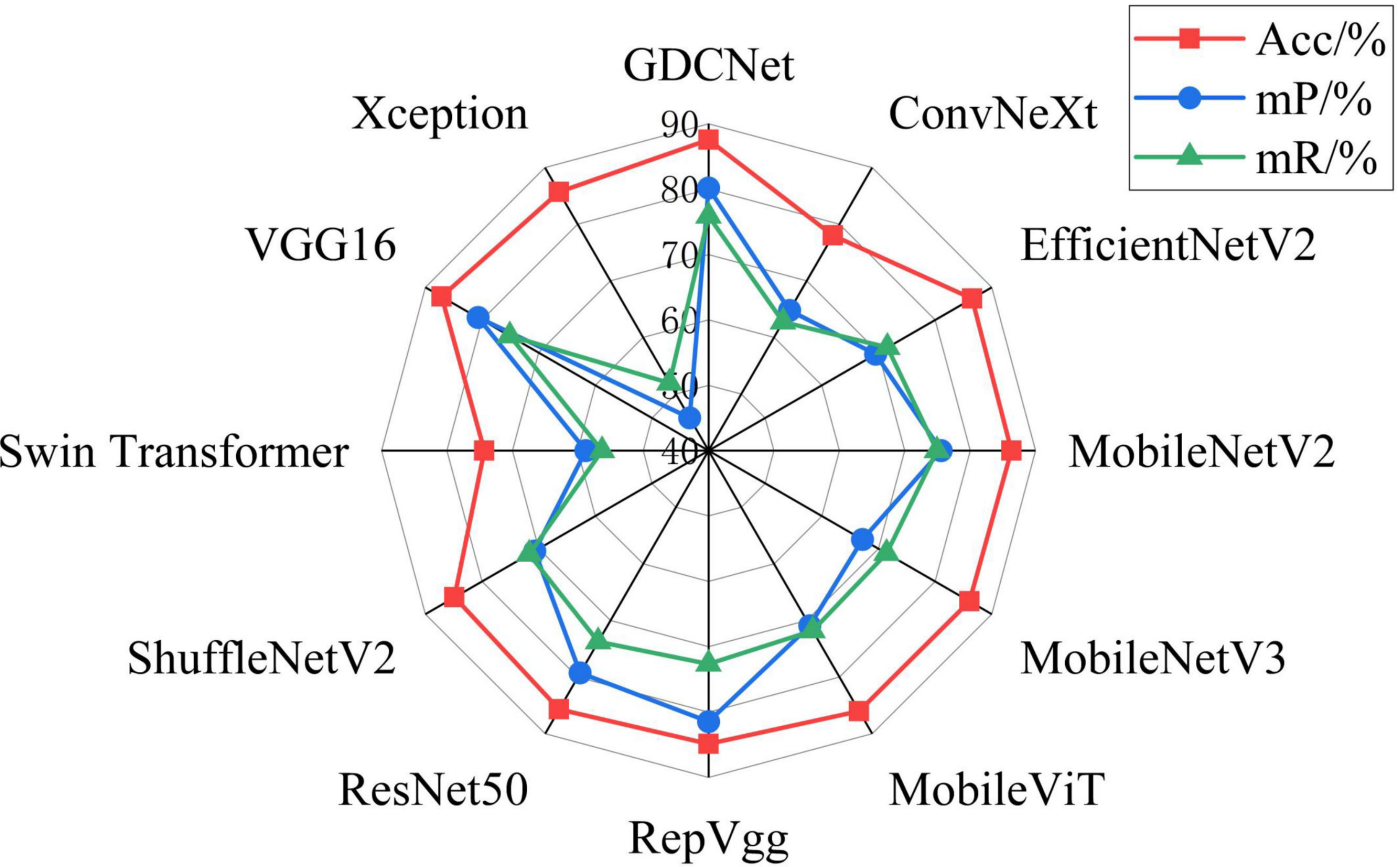
Radar chart of Acc, mP, and mR for twelve network segmentation models.

To evaluate the subgraph recognition capability of the proposed model on grape downy mildew lesions, we conducted a comparative analysis of twelve vision models across nine semantic categories using standard metrics: Acc, mP, and mR. The detailed results are shown in **Tables 6** and **Figure 6**, **7**. The K-CNN-VC segmentation outcomes are shown in **Figure 8**. **Figure 6** presents a radar chart conducting a multidimensional comparative analysis of 12 deep learning models across three core performance metrics: accuracy, mean precision, and mean recall. The results demonstrate that GDCNet achieves the highest performance in terms of accuracy, while also maintaining competitively high levels in both mean precision and mean recall, exhibiting a marked overall performance advantage. The remaining models display distinct distribution characteristics across these three metrics, visually reflecting their performance disparities and specific strengths or weaknesses in the target task.

**Table 6:** Comparison of recognition accuracy for twelve Types of model subgraphs.

Model	0/%	1/%	2/%	3/%	4/%	5/%	6/%	7/%	8/%
ConvNeXt_T	95.65	82.22	65.55	73.08	61.29	91.67	63.6	50.00	0
EfficientNetv2_S	98.09	82.89	86.78	92.52	70.07	84.21	53.85	57.14	0
MobileNetV2	95.99	78.47	93.77	86.61	82.24	80.95	50.00	61.90	50.00
MobileNetV3_S	95.50	93.62	84.64	83.46	67.18	88.89	41.38	50.00	0
MobileViT	98.49	83.16	91.03	90.24	55.84	77.78	69.23	72.73	0
RepVgg	98.99	77.61	88.15	78.18	7000	93.33	60.00	66.67	100
ResNet50	98.49	88.10	87.42	78.57	60.49	73.68	60.00	66.67	100
ShuffleNetV2	98.49	89.63	79.66	88.57	60.00	73.68	58.82	87.50	0
Swin_Transformer	97.51	59.65	90.68	58.82	48.08	86.67	54.55	10.53	0
VGG16	98.99	88.10	85.21	75.00	71.88	86.67	60.00	75.00	85.71
Xception	88.00	89.56	88.89	76.27	69.44	0	0	0	0
GDCNet	95.65	90.96	89.31	76.92	64.47	93.33	73.33	87.50	50.00

**Figure 7:**
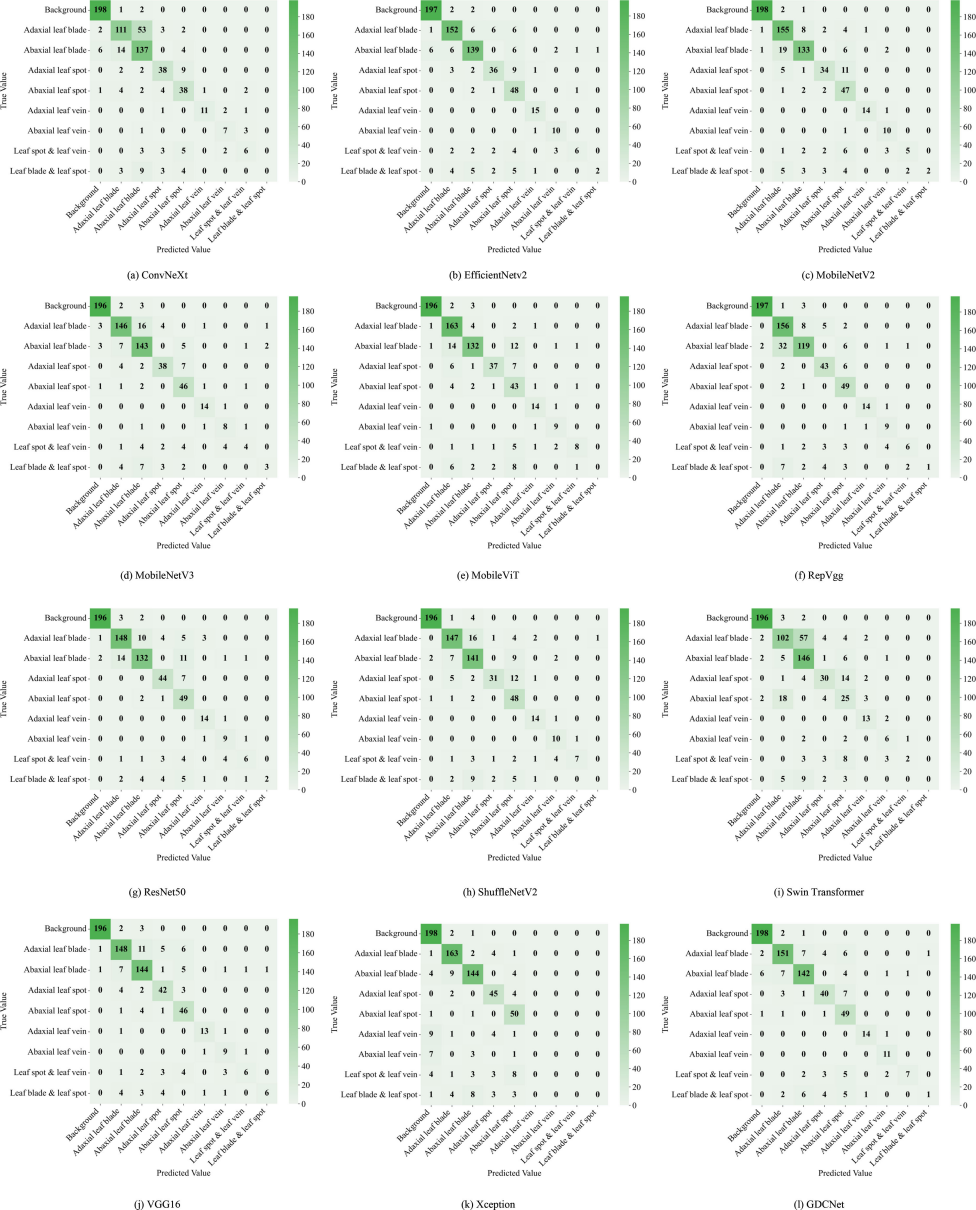
Twelve types of network segmentation confusion matrices. **(a)** illustrates the segmentation confusion matrix of the ConvNeXt network; **(b)** illustrates the segmentation confusion matrix of the EfficientNetV2 network; **(c)** illustrates the segmentation confusion matrix of the MobileNetV2 network; **(d)** illustrates the segmentation confusion matrix of the MobileNetV3 network; **(e)** illustrates the segmentation confusion matrix of the MobileViT network; **(f)** illustrates the segmentation confusion matrix of the RepVgg network; **(g)** illustrates the segmentation confusion matrix of the ResNet50 network; **(h)** illustrates the segmentation confusion matrix of the ShuffleNetV2 network; **(i)** illustrates the segmentation confusion matrix of the Swin Transformer network; **(j)** illustrates the segmentation confusion matrix of the VGG16 network; **(k)** illustrates the segmentation confusion matrix of the Xception network; **(l)** illustrates the segmentation confusion matrix of the GDCNet network.

**Figure 8:**
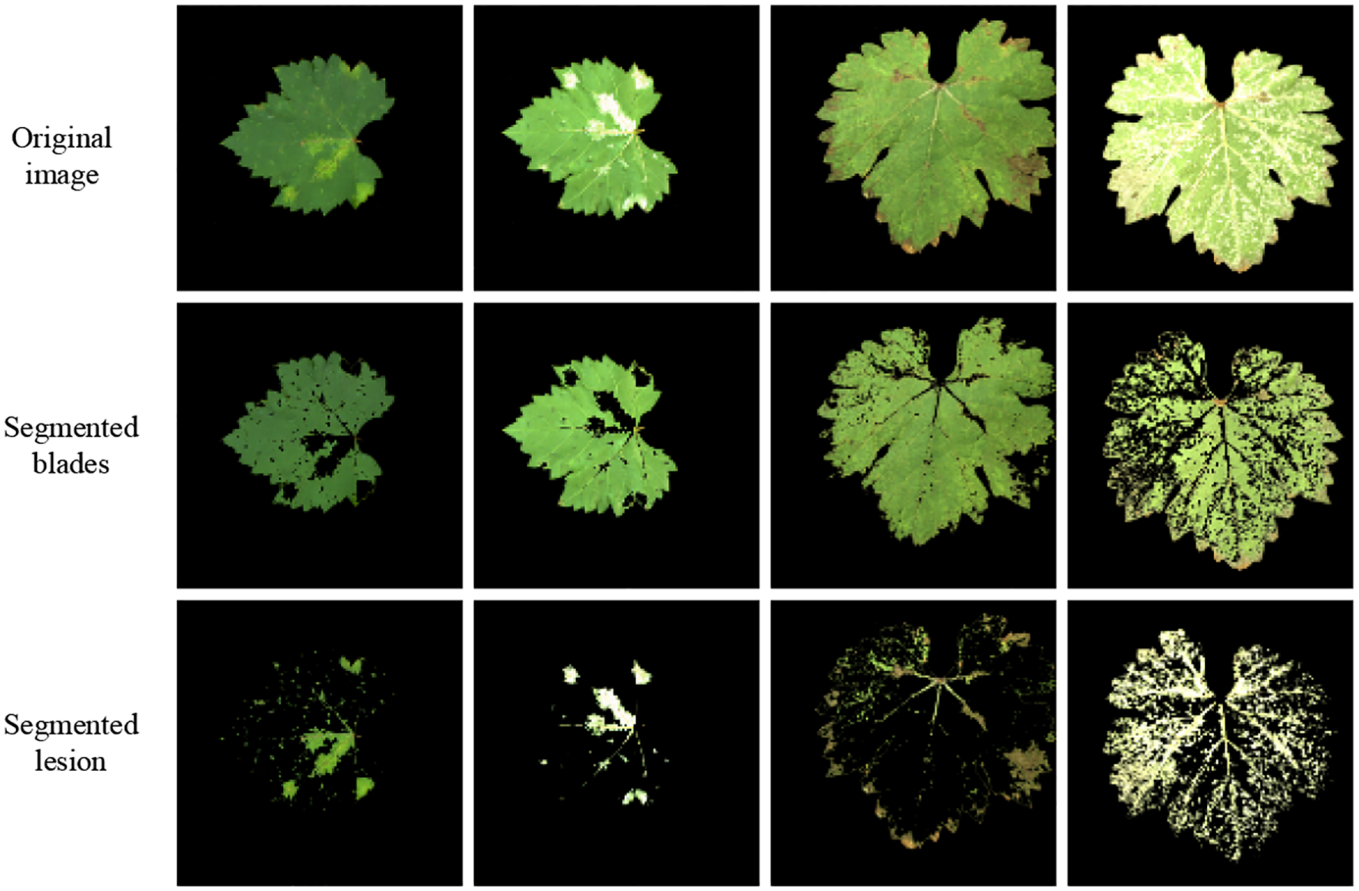
K-CNN-VC segmentation result diagram.

Among all models, MobileViT demonstrated superior performance in identifying abaxial leaf surfaces, adaxial lesions, and lesions with veins. This effectiveness can be attributed to its hybrid architecture, which combines the global modeling of Vision Transformers with the spatial local feature extraction of convoluted networks. This likely captures cross-regional dependencies in leaf textures and lesion morphologies. VGG16, a deep convolutional architecture, exhibited high accuracy for background adaxial leaf surfaces, and leaves with lesion categories, but underperformed in categories involving abaxial lesions and veins, highlighting its limitations in capturing fine-grained, low-contrast structures on the leaf underside. Similarly, Swin_Transformer struggled with lesions and veins, potentially due to its local window-based attention strategy, which led to contextual fragmentation in overlapping semantic regions.

GDCNet consistently ranked among the top performers, achieving 87.57% accuracy and 80.17% mP, while maintaining a balanced performance across both structural (for example, adaxial/abaxial veins) and composite categories (such as lesions with veins), indicating enhanced sensitivity to disease-specific traits. RepVgg and ResNet50 demonstrated strong performance in terms of mean recall and precision, benefiting from residual connections and reparameterization that enhance feature robustness in complex backgrounds. Xception achieved zero accuracy in vein-related categories, indicating that its depthwise separable convolution structure is unable to integrate fine-grained multimodal cues.

Model-task compatibility significantly affects detection performance. For example, MobileNetV2 performed well in simpler categories (such as abaxial leaf surfaces and adaxial lesions) via depthwise separable convolutions, but underperformed in composite categories (such as lesions with veins). ConvNeXt_T, while effective in recognizing adaxial veins, revealed semantic discrimination weaknesses in more nuanced or overlapping lesion types. These findings underscore that model-task compatibility has a significant influence on segmentation success. GDCNet, RepVgg, and VGG16 emerge as strong candidates for practical deployment in grape downy mildew detection, while transformer-based models require further adaptation to handle multiscale, overlapping semantic features.

### 4.3 Inversion results and analysis

#### 4.3.1 Lesion classification and feature thresholds

Contour Separation: Edge detection was applied to segmented adaxial leaf and lesion images to extract leaf and lesion edge contours. The following parameters were calculated.

Contour Perimeter (P)​: Computed using OpenCV’s arcLength function.

Enclosed Area (A)​: Computed using OpenCV’s contourArea function.

Based on characteristics of the three lesion types:

1. Dot-shaped lesions: Dense and minute; characterized by small perimeters and areas.
2. Patch-shaped lesions: Larger perimeters and areas.
3. Large-scale lesions: Variable perimeter and area (typically high values).

Parameter thresholds were set as shown in **Table 7**.

**Table 7:** Thresholds for lesion perimeter and area classification.

Type of lesion	Perimeter threshold/P	Area threshold/A
Dot-shaped lesion	P<100	A<100
Patch-shaped lesion	100<=P<400	100<=A<2000
Large-scale lesion	P>=400	A>=200

**Figure 9:**
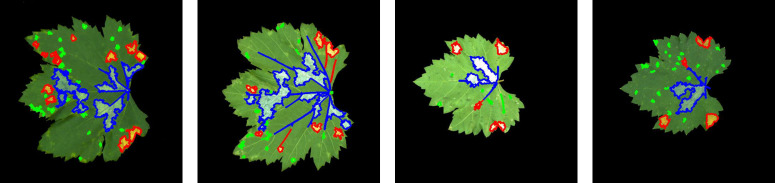
​Images of three types of lesions.

The classification results, based on the thresholds defined in **Table 7**, are presented in **Figure 9**. In ​**Figure 9**, green indicates typical ​dense and minute dot-shaped lesions, red denotes typical ​patch-shaped lesions, and blue denotes typical ​large-scale lesions.

#### 4.3.2 Adaxial–Abaxial lesion morphology mapping

**Figure 10:**
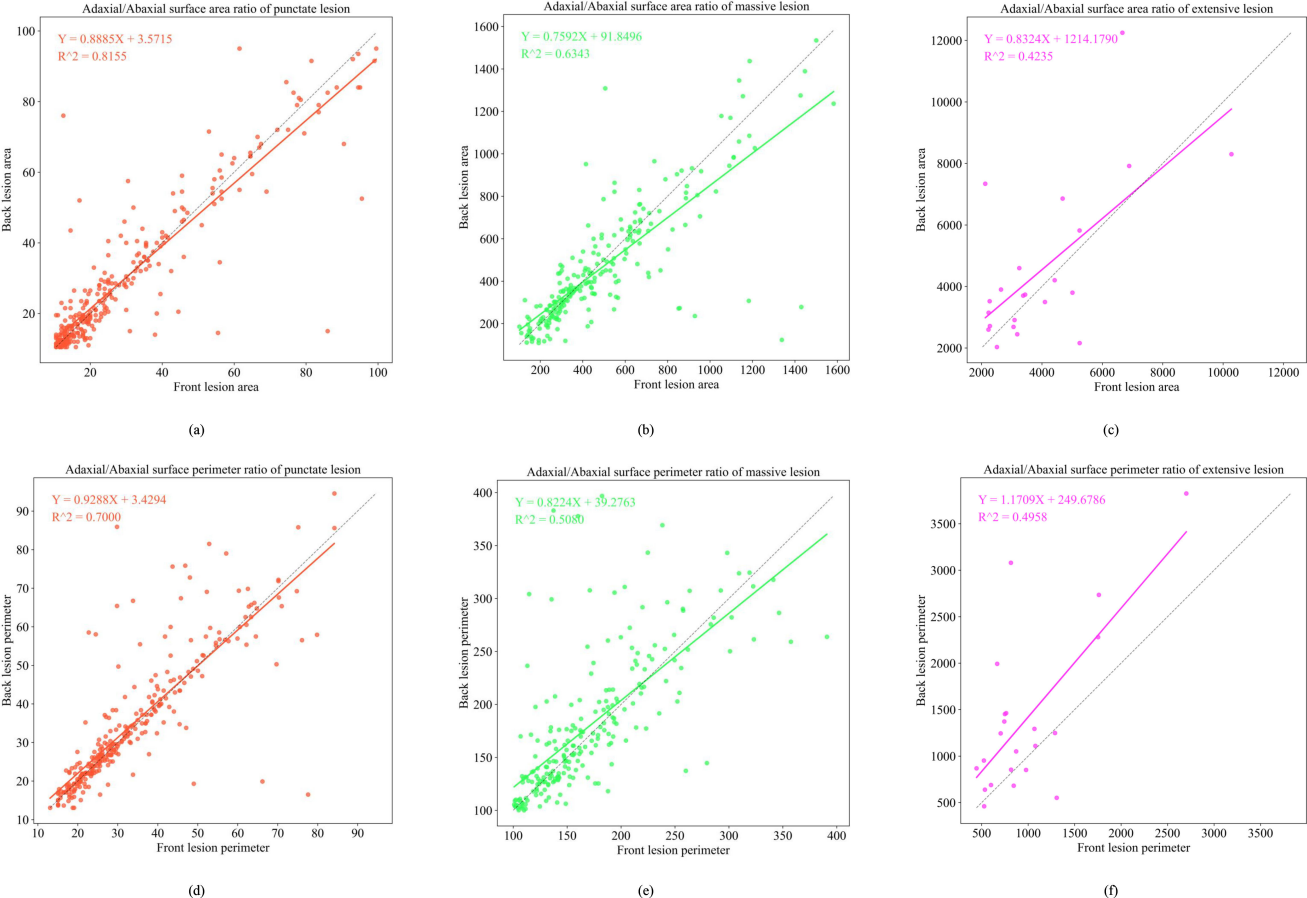
Functional relationship diagram of the front and back sides of three early-stage lesion types. **(a)** depicts the adaxial/abaxial surface area ratio of punctate lesion; **(b)** depicts the adaxial/abaxial surface area ratio of massive lesion; **(c)** depicts the adaxial/abaxial surface area ratio of extensive lesion; **(d)** depicts the adaxial/abaxial surface perimeter ratio of punctate lesion; **(e)** depicts the adaxial/abaxial surface perimeter ratio of massive lesion; **(f)** depicts the adaxial/abaxial surface perimeter ratio of extensive lesion.

**Figure 11:**
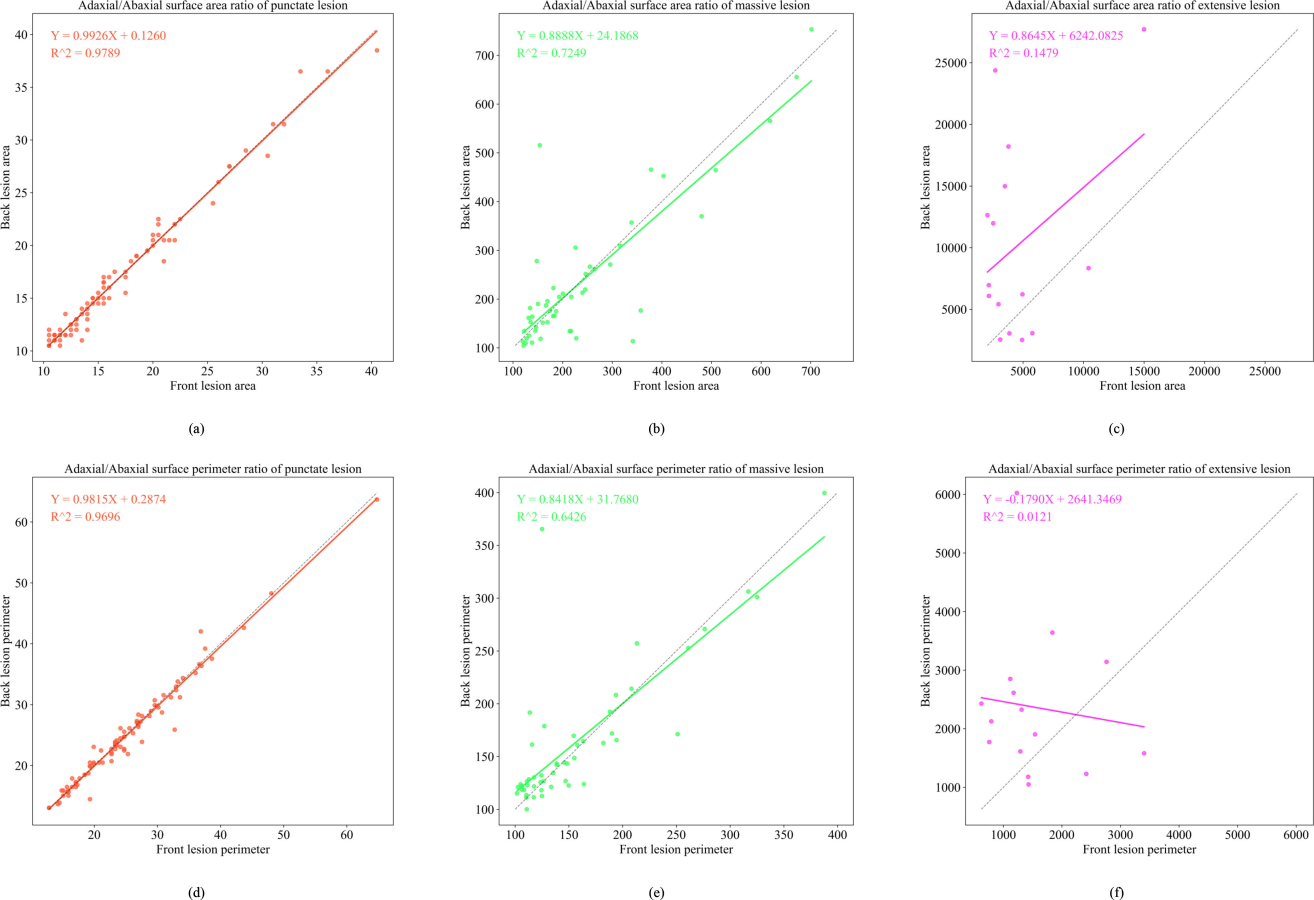
Functional relationship diagram of three types of lesions. **(a)** depicts the adaxial/abaxial surface area ratio of punctate lesion; **(b)** depicts the adaxial/abaxial surface area ratio of massive lesion; **(c)** depicts the adaxial/abaxial surface area ratio of extensive lesion; **(d)** depicts the adaxial/abaxial surface perimeter ratio of punctate lesion; **(e)** depicts the adaxial/abaxial surface perimeter ratio of massive lesion; **(f)** depicts the adaxial/abaxial surface perimeter ratio of extensive lesion.

To simulate abaxial lesion morphology from adaxial lesion characteristics, we developed regression-based mapping models for contour perimeter and area across three lesion types, as shown in **Figures 10** and **11**. In the early epidemic stage, dot-shaped lesions exhibited a linear correlation between the adaxial and abaxial areas (R^2^ = 0.8155), indicating negligible diffusion effects across the surfaces. Patch-shaped lesions show perimeter mapping with a fitness of R^2^ = 0.6343, following *Y* = 0.7592*X* + 91.8496. This reflects moderate regularity in edge expansion on the abaxial surface. Large-scale lesions exhibited poor model fit (R^2^< 0.5), primarily due to humidity-induced irregular diffusion in the abaxial microenvironment.

In the late epidemic stage, dot-shaped lesions exhibited a highly linear correlation between adaxial and abaxial areas (R^2^ = 0.9789), confirming minimal cross-surface diffusion. Patch-shaped lesions display area mapping (R^2^ = 0.7249, *Y* = 0.8888*X* + 24.1868). These values represent measurable expansion patterns on the abaxial surface. Large-scale lesions continued to exhibit low model fit (R^2^< 0.2), attributable to humidity-driven irregular spreading patterns.

#### 4.3.3 Front lesion inversion, back lesion results

**Figure 12:**
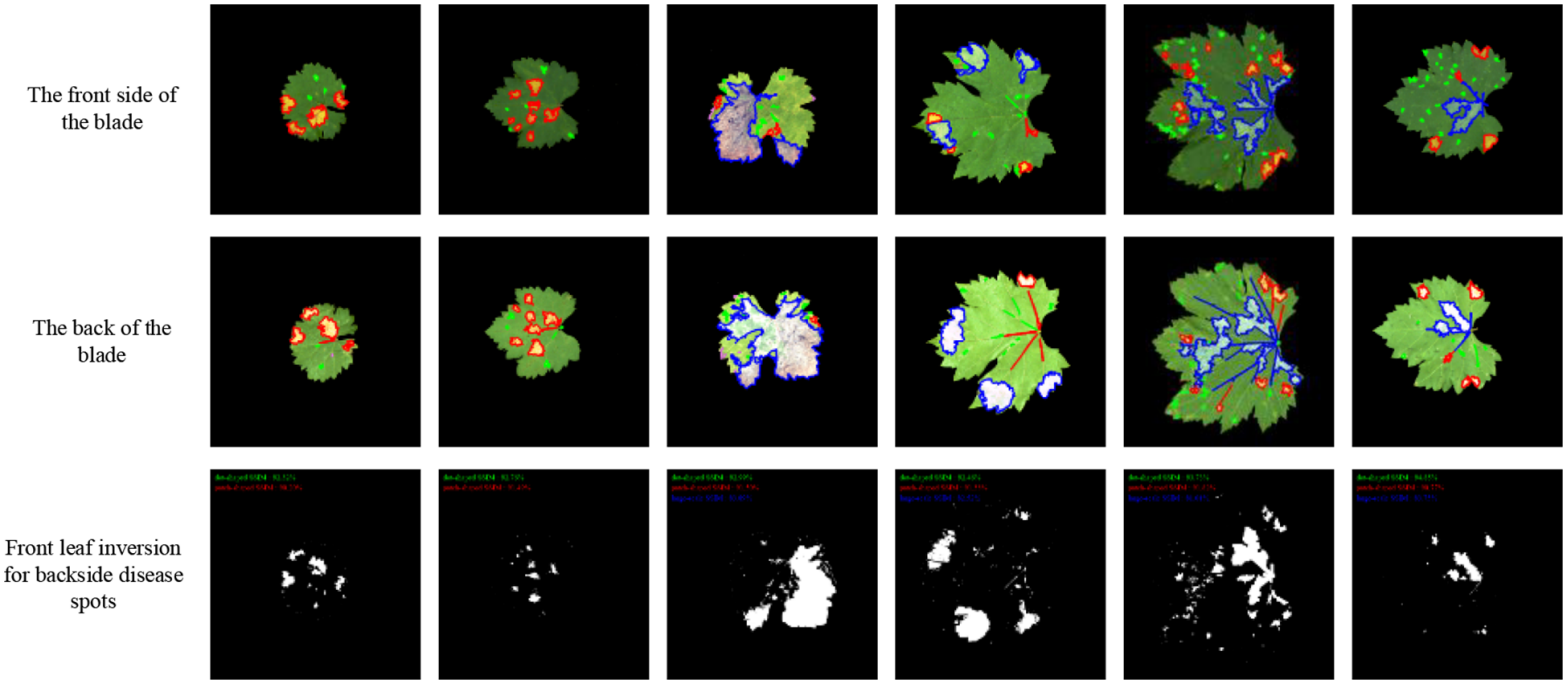
Inversion lesion result diagram.

Following the establishment of morphological mapping models, the inversion algorithm was implemented by applying dilation or erosion operations to adaxial lesion contours to generate abaxial lesion morphologies based on the perimeter and area correspondence rules derived from late-stage infection data. ​**Figure 12** shows that the average similarity between the inverted and actual abaxial lesions reached ​80%​, with an inversion accuracy exceeding ​90%​​ for ​dot-shaped ​and ​patch-shaped lesions.

## 5 Discussion

This study introduces a novel framework for grading the severity of grape downy mildew and performing adaxial-to-abaxial lesion inversion. For grading tasks, the GDCNet model achieved a lightweight and high-precision balance through the synergistic design of a cross-receptive-field fusion module (SCDCA) and a CA mechanism (CA). With a model size of 5.08 MB, GDCNet achieved a fine-grained classification accuracy of 82.16%. Conversely, the improved ResNet50 model achieved 99.92% accuracy for four broad infection stages (health/pre/mid/late-infection classification). Our model maintained fine-grained grading capability while reducing computational complexity by 2.44 GFLOPs and boosting inference speed to 0.56 ms/frame. This model is better suited for edge-computing deployment in field conditions, addressing Kumar et al.’s challenge of balancing feature representation and efficiency in lightweight models (Kumar and Sachar, 2023).

Regarding lesion segmentation, the proposed K-CNN-VC approach addresses the challenge of accurately annotating lesions with blurred boundaries, a limitation identified by Tardif et al. (2023). By integrating unsupervised clustering and supervised classification cascading, the method attains 89.29% segmentation accuracy, outperforming Li et al (2022) traditional ML method (which has 63.32% fine-grained accuracy), while avoiding the annotation burden typically associated with pixel-wise supervised models such as U-Net (Xue, 2023) These findings support the argument of Ahmad et al. (2023) that automating lesion extraction while minimizing manual effort is essential for smart agriculture applications.

This study also pioneers a morphology inversion mechanism from abaxial leaf images, marking a first in plant disease modeling literature. This approach reflects the biological propagation behavior of downy mildew. The pathogens primarily invade through stomata, propagate intercellularly within host tissues, and form lesions after a latent period (Zhang et al., 2020). Differential necrosis arises from variations in cellular ultrastructure and antioxidant enzyme activity. Based on infection patterns and lesion morphologies, we categorized the lesions into three types: dot-, patch-, and large-scale. A high inversion similarity (>90%) was observed for dot-shaped lesions, confirming uniform early infection, whereas the perimeter mapping function for patch-shaped lesions revealed medium-scale expansion patterns. Notably, poor fitting (R²< 0.5) for large-scale lesions was attributed to irregular diffusion influenced by humidity and microenvironmental variability, consistent with findings by Liu (2024) and Hernández et al. (2024) regarding necrotic patterns linked to cuticle thickness and stomatal density.

Despite promising results, the study has three key limitations:

1. The dataset includes only five North China varieties and lacks samples from peak mid-epidemic periods (August outbreak), which may limit the generalizability of the model.
2. The inversion model does not incorporate physiological parameters such as cuticle thickness and stomatal density, constraining interpretability for large-scale or atypical lesions.
3. The presence of natural lighting variations in field conditions may reduce segmentation robustness. Compared with existing approaches, such as feature-fusion-based methods (Zhang et al., 2019; Wang et al., 2023) and hybrid networks like the DualSeg network, our inversion method demonstrates robust efficacy under controlled conditions, yet its adaptability to complex backgrounds necessitates rigorous validation.
4. The abaxial lesion inversion model is primarily based on morphological feature mapping and does not consider physiological parameters such as cuticle thickness and stomatal density, which may limit its interpretability under complex or abnormal lesion conditions.

Future research should expand varietal data for cross-cultivar validation, develop dynamic pathological diffusion models to represent mid-epidemic lesion evolution, and further optimize the inversion framework by incorporating physiological descriptors, and future studies incorporate plant pathological knowledge and physiological indicators to enhance the biological plausibility of the model. Additionally, it should explore Neural Architecture Search and pruning for edge device deployment.

Such improvements will support the precision spraying paradigm aligned with China’s Grape Downy Mildew Control Standard (GB/T17980.122-2004), facilitating large-scale implementation in smart vineyard management.

## 6 Conclusion

This study presents a comprehensive framework for grape downy mildew severity grading and abaxial lesion inversion, integrating innovations in fine-grained classification, unsupervised lesion segmentation, and morphology-based inversion. Experimental results validated the effectiveness of the framework:

The GDCNet grading model, equipped with CA, achieved an accuracy of 82.16% while maintaining high inference efficiency.

The K-CNN-VC segmentation method, which combines hybrid clustering and deep learning, effectively addressed annotation ambiguity for complex lesions, achieving an accuracy of 89.29%. The morphology-based inversion model exhibited a similarity of over 80% for dot- and patch-shaped types, supporting the accurate estimation of disease severity from partial visual data. By achieving end-to-end lesion inversion from adaxial to abaxial leaf surfaces, this work lays the groundwork for automated assessment of disease progression and precision disease control. The results showed a grading accuracy of 82.29%, segmentation accuracy of 89.29%, and over 80% similarity for typical lesion types, offering key technical support for precision spraying decisions and differentiated control of grape downy mildew. Future directions should include expanding the dataset scope, enhancing biological realism in inversion, and enabling on-device deployment to support next-generation smart agricultural disease detection systems.

## Data Availability

The raw data supporting the conclusions of this article will be made available by the authors, without undue reservation.
